# The power of associative learning and the ontogeny of optimal behaviour

**DOI:** 10.1098/rsos.160734

**Published:** 2016-11-30

**Authors:** Magnus Enquist, Johan Lind, Stefano Ghirlanda

**Affiliations:** 1 Centre for the Study of Cultural Evolution, Stockholm University, Lillafrescativägen 7B, 106 91 Stockholm, Sweden; 2 Department of Zoology, Stockholm University, Svante Arrheniusvägen 14D, 106 91 Stockholm, Sweden; 3 Department of Psychology, Brooklyn College, 2900 Bedford Avenue, Brooklyn, NY 11210, USA; 4 Department of Psychology, Graduate Center of the City University of New York, 365 5th Avenue, New York, NY 10016, USA; 5 Department of Biology, Graduate Center of the City University of New York, 365 5th Avenue, New York, NY 10016, USA

**Keywords:** animal learning, optimal behaviour, reinforcement learning, conditioned reinforcement, animal cognition

## Abstract

Behaving efficiently (optimally or near-optimally) is central to animals' adaptation to their environment. Much evolutionary biology assumes, implicitly or explicitly, that optimal behavioural strategies are genetically inherited, yet the behaviour of many animals depends crucially on learning. The question of how learning contributes to optimal behaviour is largely open. Here we propose an associative learning model that can learn optimal behaviour in a wide variety of ecologically relevant circumstances. The model learns through chaining, a term introduced by Skinner to indicate learning of behaviour sequences by linking together shorter sequences or single behaviours. Our model formalizes the concept of conditioned reinforcement (the learning process that underlies chaining) and is closely related to optimization algorithms from machine learning. Our analysis dispels the common belief that associative learning is too limited to produce ‘intelligent’ behaviour such as tool use, social learning, self-control or expectations of the future. Furthermore, the model readily accounts for both instinctual and learned aspects of behaviour, clarifying how genetic evolution and individual learning complement each other, and bridging a long-standing divide between ethology and psychology. We conclude that associative learning, supported by genetic predispositions and including the oft-neglected phenomenon of conditioned reinforcement, may suffice to explain the ontogeny of optimal behaviour in most, if not all, non-human animals. Our results establish associative learning as a more powerful optimizing mechanism than acknowledged by current opinion.

## 1. Introduction

We often marvel at animals performing efficiently long sequences of behaviour, and theoretical and empirical studies confirm that animals behave optimally or near-optimally under many circumstances [1–3]. Typically, optimal behaviour has been assumed to result from natural selection of genetically determined behaviour strategies [4], yet in many species behaviour is crucially shaped by individual experiences and learning [5–7]. Existing work has considered how learning can optimize single responses [8–13] or specific sequences of two or three behaviours [14,15]. However, the question of how, and how much, learning contributes to optimal behaviour is still largely open. Here we analyse in general the conditions under which associative learning can optimize sequences of behaviour of arbitrary complexity.

Associative learning is acknowledged to contribute to adaptation by enabling animals to anticipate meaningful events (Pavlovian, or ‘classical’ conditioning) and to respond appropriately to specific stimuli (operant, or instrumental conditioning) [16,17]. Associative learning, however, is also considered mindless, outdated and too limited to learn complex behaviour such as tool use, foraging strategies or any behaviour that requires coordinating actions over a span of time (e.g. [18–21]). Such behaviour, when it is not considered genetically determined, is attributed to other learning mechanisms, usually termed ‘cognitive’ (e.g. [22–24]). Associative learning, however, has not been evaluated rigorously as a potential route to optimal behaviour [25,26]. Rather, claims about its limitations have rested on intuition rather than formal analysis and proof. In this paper, we develop an associative learning model that can be proved to closely approximate optimal behaviour in many ecologically relevant circumstances. The model has two key features: it augments standard associative learning theory with a mathematical model of conditioned reinforcement, and it integrates instinctual and learned aspects of behaviour in one theoretical framework. The latter aspect is discussed later; in this introduction, we focus on conditioned reinforcement.

Conditioned reinforcement (also referred to as secondary reinforcement) is a learning process whereby initially neutral stimuli that predict primary reinforcers can themselves become reinforcers [27–30]. For example, a dog that repeatedly hears a click before receiving food will eventually consider the click rewarding in itself, after which it will learn to perform behaviour whose sole outcome is to hear the click [31]. Conditioned reinforcement was a prominent topic in behaviourist psychology [27,29,32–34], but interest in it waned with behaviourism [35]. As a result, conditioned reinforcement was left out of the mathematical models of the 1970s and 1980s that still form the core of animal learning theory [36–40]. There are two fields, however, that have carried on the legacy of conditioned reinforcement research. The first is animal training, in which methods that rely on conditioned reinforcement are the primary tool to train behaviour sequences (see below and [31]). The second is the field of reinforcement learning, a branch of artificial intelligence that blends ideas from optimization theory and experimental psychology [41,42], and which has also become influential in computational neuroscience (e.g. [43,44]). The key element of reinforcement learning algorithms, referred to as learning based on temporal differences, is closely related to conditioned reinforcement [45–49]. A remarkable result of reinforcement learning research is that conditioned reinforcement implements a form of dynamic programming. The latter is an optimization technique used extensively by biologists to find optimal behavioural strategies, and therefore, to assess whether animals behave optimally [1,2]. It is not, however, a realistic model of how animals can *learn* to behave optimally, as it requires perfect knowledge of the environment and extensive computation. Conditioned reinforcement, on the other hand, is computationally simple as well as taxonomically widespread, suggesting that optimal behaviour may be learned rather than inherited [47].

The conceptual connections that we just summarized have been noted previously (e.g. [41,47]), but have not translated into a coherent research program. Conditioned reinforcement has not been systematically integrated with animal learning theory, nor with knowledge about instinctual behaviour from ethology, nor with the study of optimal behaviour in behavioural ecology. Our goal is to sketch a first such synthesis. We call our learning model ‘chaining’ after Skinner [30,50,51], who described how conditioned reinforcement can link together single behaviours to form sequences (chains) that ultimately lead to primary reinforcement.

## 2. Chaining: dynamic programming *in vivo*

To highlight connections to associative learning theory, behavioural ecology and reinforcement learning, we present our model in successive steps. We first consider a standard model of associative learning without conditioned reinforcement. This model can optimize single behaviours but not behaviour sequences. We then add conditioned reinforcement, obtaining our chaining model. Lastly, using ideas from reinforcement learning, we show that chaining can optimize sequences of behaviour in a similar way to dynamic programming.

Our general framework is as follows. We consider an animal that can find itself in a finite (albeit arbitrarily large) number of environmental states, among which transitions are possible. For example, states may represent spatial locations, and state transitions movement from one location to another. We assume that the animal can perceive without ambiguity which environmental states it is in (see §(c) and appendix A.3 for discussion, and for a model that does not require this assumption). By choosing its behaviour, the animal can influence transitions from one state to the next. Transitions can be deterministic (in each state, each behaviour always leads to the same next state) or stochastic (in each state, a behaviour may lead to different states, with fixed probabilities). Each state *S* has a primary reinforcement value, *u*_*S*_, which is genetically determined and serves to guide learning towards behaviour that promotes survival and reproduction. For example, a state corresponding to the ingestion of food would typically have positive value, while a state representing harm to the body would have a negative value. States that describe neutral conditions, e.g. waiting, are assumed to have a small negative value, corresponding to the time and energy expended while in the state. The animal's goal is to choose its behaviour to maximize the total value collected. To begin with, we do not assume any innate knowledge of the environment beyond the ability to recognize a number of biologically relevant situations such as pain and the ingestion of food, which are assumed to have suitable *u*_*S*_ values. Hence, the appropriate behaviour must be learned.

### 2.1. Learning a single behaviour

Consider first the optimization of a single behavioural choice. For example, we may consider a bird that finds a fruit and can choose out of a repertoire of *m* behaviours (peck, fly, sit, preen, etc.). One behaviour (peck) leads to a food reward (tasting the fruit's sweet juice); all others have no meaningful consequences. We can imagine the animal as attempting to estimate the value of each behaviour, in order to then choose the one with highest value (this notion will be made precise below). Suppose the animal is in state *S*, chooses behaviour *B* and finds itself in state *S*′. Note that, in general, a state *S* may be followed by a number of states *S*′, either because the environment is not deterministic or because the animal does not always use the same behaviour *B* when it finds itself in state *S*. Hence *S*′ does not represent a fixed state, but rather whichever state follows *S* on a particular occasion. Let *v*_*S*→*B*_ be the value estimated by the animal for choosing behaviour *B* in state *S*, and *u*_*S*′_ the primary reinforcement value of *S*′. A simple way of learning useful estimates is to update *v*_*S*→*B*_ as follows after each experience:

2.1 $$\Delta v_{S\to B}=\alpha_{v}(u_{S^{\prime }}-v_{S\to B}),$$

where Δ*v*_*S*→*B*_ is the change in *v*_*S*→*B*_, and *α*_*v*_ is a positive learning rate. The meaning of equation (2.1) is easily understood in a deterministic environment. In this case, the state *S*′ is always the same, hence *u*_*S*′_ is fixed. Over repeated experiences, equation (2.1) causes *v*_*S*→*B*_ to approach the value *u*_*S*′_. Thus, the value of choosing *B* in state *S* is equated with the primary reinforcement value that can be obtained by such a choice. If the environment is not deterministic, *v*_*S*→*B*_ approaches the average reward value of all states *S*′ that follow, each weighed by its probability of occurrence, provided *α*_*v*_ is not too large. Equation (2.1) is identical to the classic Rescorla–Wagner learning rule [36], but we consider it in an instrumental rather than a Pavlovian setting [37].

To complete our model, we need to specify how behaviours are chosen. The basic requirement for a viable decision rule is that it should preferentially choose behaviours that have a higher estimated value (so that rewards can be collected), while at the same time leaving some room for exploring alternative behaviours (so that accurate value estimates can be learned). A simple way to address both concerns is the so-called ‘softmax’ rule, which specifies the probability of behaviour *B* in state *S* as:

2.2 $$\mathrm{Pr}(S\to B)=\frac{e^{\beta v_{S\to B}}}{\sum_{B^{\prime }}e^{\beta v_{S\to B^{\prime }}}},$$

where the sum runs over all possible behaviours. The parameter *β* regulates exploration: if *β*=0 all behaviours are equally likely irrespective of estimated value, whereas if *β* is very large only the behaviour with the highest estimated value occurs with any likelihood. Equation (2.2) is broadly compatible with known aspects of animal choice behaviour. For example, if two behaviours *B*_1_ and *B*_2_ have different estimated values in state *S*, equation (2.2) does not choose exclusively the more profitable one. Rather, the relative probability of choice depends on the difference in estimated values:

2.3 $$\frac{\mathrm{Pr}(S\to B_{1})}{\mathrm{Pr}(S\to B_{2})}=\frac{e^{\beta v_{S\to B_{1}}}}{e^{\beta v_{S\to B_{2}}}}=e^{\beta (v_{S\to B_{1}}-v_{S\to B_{2}})}.$$

This relative preference is compatible with the ‘matching law’ of experimental psychology, according to which the probability of choosing a behaviour is an increasing function of the amount of reinforcement obtained from the behaviour [52,53].

### 2.2. Learning behaviour sequences

**Figure 1.**
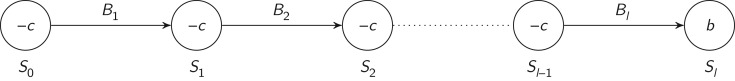
A simple environment in which a sequence of *l* actions is required in order to reach a reward. The animal can be in any of *l*+1 states, numbered 0 to *l* and represented as circles. Numbers inside the circles represent primary reward values (*u*_*S*_). The last state has positive value; other states have negative value (*b*,*c*>0). In each state, the animal can choose a behaviour from a repertoire of *m* behaviours. In each state there is a ‘correct’ behaviour that brings the animal to the next state (shown by arrows). All other behaviours bring the animal back to state 0 (not shown to avoid clutter), at which point the animal can attempt again to reach the rewarding state *l*. When state *l* is reached, the animal goes back to state 0 and can try again to reach the reward.

while equation (2.1) can optimize a single behavioural choice, it cannot optimize sequences of behaviours. Figure 1 shows a simple environment in which the animal has to perform correctly a sequence of *l* actions in order to reach a reward. Equation (2.1) can learn the correct behaviour in state *l*−1 because it results in a reward. In the other states, however, correct behaviours are not rewarded, and equation (2.1) will learn to assign them a value of −*c*, i.e. the same value as the incorrect behaviours. The problem can be overcome if states can acquire conditioned reinforcement value. For example, if the animal repeatedly chooses the correct action in state *l*−1 and thereby experiences the rewarding state *l*, then state *l*−1 will acquire conditioned reinforcement value. Conditioned reinforcement functions in the same way as primary reinforcement. That is, if the animal now takes the correct action in state *l*−2 and thereby transitions in state *l*−1, then state *l*−1 will be deemed reinforcing. Thus taking the correct action will be reinforced; additionally, state *l*−2 will in turn acquire conditioned reinforcement value. In this way, value can backtrack all the way to the beginning of the chain and eventually reinforce correct actions even in state 0. We now formalize these intuitions.

Let *w*_*S*_ be the conditioned reinforcement value of state *S*. It is natural to modify equation (2.1) as follows:

2.4 $$\Delta v_{S\to B}=\alpha_{v}(u_{S^{\prime }}+w_{S^{\prime }}-v_{S\to B}).$$

In other words, the value of behaviour *B* in state *S* is taken to be the sum of the primary and conditioned reinforcement values of state *S*′. In this way, reaching a state with conditioned value can be reinforcing, even if the state has no primary value. But how do states acquire conditioned value? We assume that the conditioned value of *S* is updated according to:

2.5 $$\Delta w_{S}=\alpha_{w}(u_{S^{\prime }}+w_{S^{\prime }}-w_{S}),$$

where Δ*w*_*S*_ is the change in *w*_*S*′_ and *α*_*w*_ a positive parameter akin to *α*_*v*_ in equation (2.4). According to equation (2.5), the conditioned reinforcement value *w*_*S*_ is updated to approach the value *u*_*S*′_+*w*_*S*′_, i.e. the total value of the following state. We continue to assume that decision making operates according to equation (2.2). Equations (2.4) and (2.5) constitute our chaining model. They appear in [54] in the context of machine learning, but have been used only sporadically in this field. They also appear in [15] without justification. In the next section, we provide two examples of how chaining can relate to animal learning, and in the following section we discuss how chaining can learn optimal behavioural strategies.

### 2.3. Two examples: self-control and expectations

To illustrate our model, and substantiate the claim that associative learning is currently underestimated, we consider two ‘cognitive’ phenomena, self-control and expectations of future events, that are commonly thought to lie beyond the scope of associative learning [55,56].

**Figure 2.**
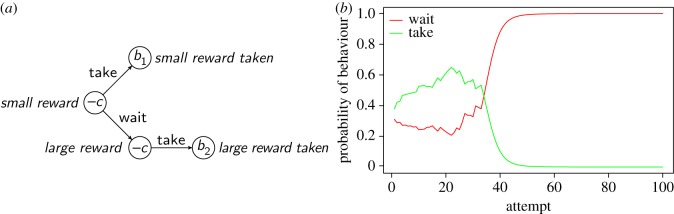
Self-control through chaining. (*a*) A task in which the animal can either take a small reward immediately, or wait and take a larger reward later. Each circle is a state with its value inscribed. We set *c*=0.2, *b*_1_=1 and *b*_2_=5. In all states, a third action (not represented) causes the animal to leave the task and go back to the initial state (*Small reward*). (*b*) Sample simulation of the chaining model on this task. An ‘attempt’ (horizontal axis) is defined as a sequence of actions comprising two successive visits to the initial state. Model parameter values where *α*_*v*_=0.1, *α*_*w*_=0.1 and *β*=2.

Many species, both in the laboratory and in nature, demonstrate a degree of self-control, ‘the ability to inhibit a pre-potent but ultimately counter-productive behaviour’ [56]. At first sight, associative learning would seem to always prefer an immediately rewarded response to a non-rewarded one, which would result in poor self-control [55]. However, ‘wait’ is also a behaviour that can be reinforced [31]. Figure 2, indeed, shows that self-control can be learned through chaining in a task similar to those used in the literature on self-control [56]. In a representative simulation, we see that waiting initially struggles to increase in frequency, but eventually it is learned as the most profitable option. Functionally, this is simply a consequence of the optimizing power of chaining: it can learn to forfeit an immediate reward for a future one given that it is optimal to do so. Mechanistically, waiting can be reinforced if it results in stimuli that are conditioned reinforcers. This is what happens in our simulation: the correct sequence (wait, then take) is initially performed by chance, which leads to the intermediate state acquiring conditioned reinforcement value. At this point, waiting can be reinforced and taking the small reward is progressively abandoned.

Chaining, however, can fail to learn self-control if the animal cannot distinguish between states in which it pays to wait and states in which it pays to act, or if the benefit of waiting is not experienced. These considerations may help explain why many animals find it hard to postpone rewards (e.g. [57]). Such ‘impulsivity’ is not necessarily maladaptive: it often pays a cheetah to wait upon sighting a prey, but it seldom pays a toad. Differences in self-control can result from genetic evolution tuning learning mechanisms to each species' environment (see below and [58,59]). For example, cheetahs may have a genetic predisposition for waiting rather than attacking immediately, which would facilitate the discovery that waiting is profitable. One way to build such a predisposition into our model is to let the *β* value for waiting be higher than that for attacking, in states that correspond to a prey having been spotted. Such a difference in *β* would lead to waiting being chosen more often in these states. A high initial value of *v*_*S*→*B*_ in these states would also make waiting more likely.

The view that self control is learned based on a genetic predisposition for waiting is consistent with the observation that self-control correlates with absolute, but not relative brain size [56]. The latter is often considered a proxy for cognitive sophistication, while the former correlates with body size and lifespan. Hence a possible reading of the data is that longer lived animals have more self-control, which is expected as they have more time to invest in learning longer behaviour sequences [60,61]. Thus taxonomic variation in self-control may result from tuning chaining to the needs of different species, rather than from different species having a more or less developed ‘cognitive ability’ for self-control.

Similar arguments apply to expectations. Animals often show ‘expectations’ about forthcoming events. For example, they may react to the omission of an expected food reward by searching for the missing food, or with aggressive behaviour [62,63]. Search is appropriate when food is likely to have disappeared out of sight, whereas aggression is suitable when food is likely to have been stolen. At first sight, such behaviour seems hard to reconcile with associative learning, including chaining, because these mechanisms do not formulate explicit predictions about forthcoming events (e.g. [16,64]). Associative learning models, however, do compute the extent to which the reinforcement value of stimuli is ‘surprising’ or ‘unexpected’ [36,65]. Our model, for example, calculates differences between estimated and realized values, such as equation (2.4). For brevity, let us write the error term in equation (2.4) as

2.6 $$d_{v}=u_{S^{\prime }}+w_{S^{\prime }}-v_{S\to B.}$$

A negative *d*_v_ means a smaller reward than expected, whereas a positive *d*_v_ indicates a larger reward. The usual role of differences such as *d*_v_ in animal learning theory is to drive learning (see equation (2.4)), but they have also been suggested to influence choice of behaviour [28,66,67]. Animals may have genetic predispositions that favour certain behaviours when *d*_v_ signals a violated expectation. Formally, we can let *d*_v_ influence the value of *β* in equation (2.2). For example, setting *β*=*β*_0_−*d*_v_ (where *β*_0_ is a baseline value) for aggressive behaviour will make aggression more likely when *d*_v_<0, i.e. when an expected reward is omitted. (Aggression would also be less likely when *d*_v_>0, e.g. when a reward is larger than expected.) This assumption is consistent with the observation that larger violations of expectations trigger more pronounced responses [62].

### 2.4. Learning optimal behaviour

We derived equations (2.5) and (2.4) by adding conditioned reinforcement to a standard model of associative learning. The same equations can be derived from considerations of optimality based on work from reinforcement learning [41]. This derivation shows how chaining is connected to dynamic programming and optimization. Consider a task that is guaranteed to last a finite time, and let $w_{S}^{*}$ be the expected reward that can be gained from all states that come after state *S*, when following a given behavioural strategy (in our case, equation (2.2) with a given set of *v*_*S*→*B*_ values). Formally

2.7 $$w_{S}^{*}=E_{S}\left(\sum_{X=S^{\prime }}^{S_{\mathrm{end}}}u_{X}\right),$$

where the sum runs on successive states, *S*′ being the state that follows *S*, and *S*_end_ the state that ends the task. *E*_*S*_(⋅) is the expectation with respect to all possible successions of states, from *S* until the end of task. We have an expectation rather than a fixed number because both the task and the behavioural strategy may not be deterministic, so that many possible sequences of states and actions are possible starting from state *S*. In equation (2.7), the first term in the sum is *u*_*S*′_:

2.8 $$\begin{array}{rl} w_{S}^{*} & =E_{S}\left(u_{S^{\prime }}+\sum_{X=S^{\prime \prime }}^{S_{\mathrm{end}}}u_{X}\right) \end{array}$$

2.9 $$\begin{array}{rl} & =E_{S}(u_{S^{\prime }})+E_{S}\left(\sum_{X=S^{\prime \prime }}^{S_{\mathrm{end}}}u_{X}\right). \end{array}$$

If *P*_*S*,*S*′_ is the probability to go from *S* to *S*′, the first expectation is simply

2.10 $$E_{S}(u_{S^{\prime }})=\sum_{S^{\prime }}P_{S,S^{\prime }}u_{S^{\prime }}$$

because *u*_*S*′_ depends only on *S*′ but not on later steps. In the second expectation, we can also make explicit this first step:

2.11 $$E_{S}\left(\sum_{X=S^{\prime \prime }}^{S_{\mathrm{end}}}u_{X}\right)=\sum_{S^{\prime }}P_{S,S^{\prime }}E_{S^{\prime }}\left(\sum_{X=S^{\prime \prime }}^{S_{\mathrm{end}}}u_{X}\right)$$

and note that, by the definition in equation (2.7), the remaining expectation is $w_{S^{\prime }}^{*}$, where *S*′′ is the state that follows *S*′. We can thus rewrite equation (2.7) as:

2.12 $$w_{S}^{*}=\sum_{S^{\prime }}P_{S,S^{\prime }}(u_{S^{\prime }}+w_{S^{\prime }}^{*}).$$

This is a necessary consistency condition that $w_{S}^{*}$ values must satisfy in order to represent the reward expected after state *S* [41]. Equation (2.12) expresses the fact that the reward expected after state *S* is the reward expected from the next state, $\sum_{S^{\prime }}P_{S,S^{\prime }}u_{S^{\prime }}$, plus the reward expected from all following states, which must equal $\sum_{S^{\prime }}P_{S,S^{\prime }}w_{S^{\prime }}^{*}$ by definition of $w_{S}^{*}$. In this way, $w_{S}^{*}$ values take into account long-term outcomes in addition to immediate reward. Equations such as (2.12) are referred to as Bellman equations and are the foundation of dynamic programming [41,68]. As we recalled in the introduction, dynamic programming is a useful computational tool to find optimal behavioural strategies, but it does not explain how animals may learn such strategies. Crucially, however, we can see that chaining performs approximate dynamic programming during an animal's lifetime. Indeed, from equation (2.5) we can calculate the expected change in *w*_*S*_ over one step of the dynamics:

2.13 $$E(\Delta w_{S})=\sum_{S^{\prime }}P_{S,S^{\prime }}\Delta w_{S}=\alpha_{w}\left(\sum_{S^{\prime }}P_{S,S^{\prime }}(u_{S^{\prime }}+w_{S^{\prime }})-w_{S}\right).$$

In the parenthesis, we can now recognize an approximation to $w_{S}^{*}$, obtained by replacing the true value of the next state ($w_{S^{\prime }}^{*}$) with its conditioned reinforcement value (*w*_*S*′_), which has been learned through experience. Thus, over successive passes through *S*, equation (2.5) works to reduce the difference between the current value of *w*_*S*_ and an estimate of its true expected value. A similar argument can be made for equation (2.4). In appendix A, we show that this process is expected to eventually approximate closely the true $w_{S}^{*}$ and $v_{S\to B}^{*}$ values (the latter being the long-term reward expected after choosing *B* in *S*). Thus, chaining can behave optimally within the limits of exploration given by equation (2.2). In practice, a requirement for convergence is that *α*_*w*_ and *α*_v_ be small enough that accumulating changes in *w*_*S*_ and *v*_*S*→*B*_ over successive experiences approximates the averaging operation in equation (2.13) and the analogous equation for *v*_*S*→*B*_.

## 3. Efficiency of chaining

We established above that chaining is expected to learn optimal behaviour sequences, but we did not discuss how long that may take. In this section, we delimit the conditions under which chaining can learn within a realistic time frame. We start with the task in figure 1, followed by variants that apply to different learning scenarios.

### 3.1. The basic learning time

In the task in figure 1, an animal has to learn a sequence of *l* behaviours, each of which can be chosen from a repertoire of *m* behaviours. At each step, only one action is correct, resulting in progression to the next step; all other actions cause the animal to have to start from scratch. Although chaining can learn the task, the expected learning time is at least *m*^*l*^ attempts (see appendix), implying that even moderately long sequences may not be learned realistically. A sequence of four behaviours drawn from a repertoire of just 10 behaviours is expected to take 10^4^=10 000 attempts to learn. The fundamental reason for such long learning times is that chaining (or any other learning mechanism) is not magic: it still needs to find the reward, which initially can happen only by chance. Another factor complicates the problem further in natural environments. Imagine an inexperienced predator that can choose to hunt either a small prey that can be caught with a single behaviour, or a large prey that can be caught through a sequence of behaviours. The predator may be initially unbiased in its choice, yet hunting the small prey will be rewarded much more often than hunting the large one. Thus, the predator may end up hunting the small prey more and more often, making it less and less likely that it will practise with hunting the large prey. In general, the possibility of choosing between easy and hard problems weighs against learning the hard problems, because the learner can obtain rewards on easy problems that will cause it to spend less time exploring the hard ones.

In summary, it is important to recognize that, although chaining can learn arbitrarily long sequences of behaviours, in practice, it cannot do so within a reasonable time *unless favourable circumstances intervene*. Understanding what these circumstances may be is crucial in evaluating whether chaining can plausibly account for the ontogeny of optimal behaviour. One favourable circumstance is having some knowledge of the task, so that not everything has to be learned. We will discuss below how this can be accomplished through genetic predispositions that favour appropriate behaviour. Another circumstance leading to shorter learning times does not depend on prior knowledge, but rather on the environment facilitating task solution, as we discuss next.

### 3.2. Entry and exit patterns

The goal of this section is to show that learning times can be dramatically shorter than what we just calculated, provided a task is sometimes entered from states that are close to the reward. For example, a squirrel that is learning to handle hazelnuts may occasionally find one with a broken or weakened shell, which would reinforce the final prying behaviour without the need to master biting and cracking the shell. In §(a), we will consider other examples.

We call the rules that govern how often each state is entered the ‘entry pattern’ of a task. For the sake of illustration, we consider the model task in Figure 1 with the following family of entry patterns:

— With probability *p*, the animal enters the task at a random state (excluding the last, rewarded state).
— With probability 1−*p*, the animal enters at the first state.

**Figure 3.**
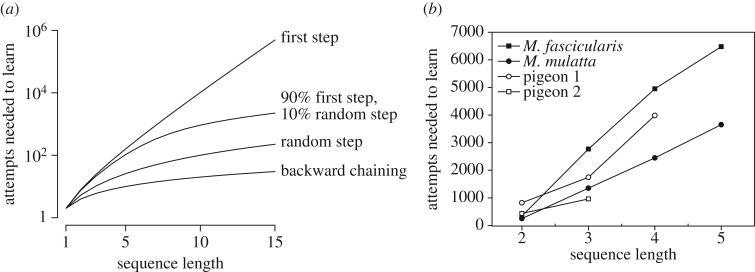
(*a*) Graph of some of the equations in table 1, *a*=1 and *m*=2. Note the logarithmic vertical axis. (*b*) Number of attempts required to learn sequences of different length trained by forward chaining, supporting the linear relationship expected under the hypothesis that chaining is the mechanism underlying such learning. Learning criteria was 80% correct in the macaque studies [69], 70% in pigeon 1 (only 5 of 7 pigeons learned the four-step sequence [70]) and 75% for pigeon 2 [71]. The behaviour sequence consisted of pressing response keys in a specific order.

We continue to assume that all mistakes bring the animal back to the first state. Setting *p*=0 means always entering at the first state. In this case, the learning time is exponential in the length of the sequence, as discussed above. As *p* increases, entering at states that are closer to the reward becomes more likely. Figure 3*a* shows the dramatic effect that even a moderate facilitation, *p*=0.1, has on learning times. Thus a favourable entry pattern can bring within reach a task that otherwise could not be learned in a realistic time frame. If *p*=1 (entry at a random state), the learning time is only quadratic in the length of the sequence (table 1 and appendix).

**Table 1.** Learning times of the chaining model as a function of ‘entry patterns’, i.e. ways in which an animal may enter a task to be learned. Symbols: *l*, sequence length; *m*, number of behaviours available at each step; *a*, number of trials required to learn a rewarded action. Note that forward chaining is not purely an entry pattern as it includes rewarding intermediate states, see text.

learning situation	entry pattern	learning time	
animal on its own	start	exponential	∼*am*^*l*^
animal on its own	random	quadratic	$∼\frac{am}{2}l(l+1)$
animal on its own or backward chaining arranged by trainer	highest state not yet learned	linear	∼*alm*
forward chaining arranged by trainer	start	linear	∼*alm*

Animal trainers exploit favourable entry patterns to teach remarkably long sequences of behaviour. In *backward chaining* (where ‘chaining’ refers to the training technique rather than to a learning process), sequences are taught starting from the last step. That is, the animal is placed repeatedly at state *l*−1 of an *l*-step sequence, until the correct behaviour is learned. This also endows state *l*−1 with conditioned reinforcement value. At this point, the animal is placed in state *l*−2, where correct behaviour can be reinforced by the conditioned reinforcement value that state *l*−1 has acquired during the previous training. Once the second-last step is learned, the animal is placed in the third-last and so on. In this way, the animal is always learning just one behaviour, and the learning time becomes linear in the length of the sequence. In *forward chaining*, training starts from the first step, but the reward structure of the task is altered so that initially even performing the first behaviour is rewarded. Once the first behaviour is learned, the animal is required to perform two behaviours to obtain the reward, and so on until the whole sequence is learned. This also results in linear learning time, which agrees with observations (figure 3*b*).

Related to entry patterns are ‘exit patterns’, by which we refer to what happens when an *incorrect*behaviour is chosen. So far, we have assumed that incorrect behaviours would result in starting back from scratch. For example, a predator that makes a mistake when approaching prey will most probably lose the opportunity to catch the prey. In other cases, however, mistakes have milder consequences. A squirrel attempting to open a nut, for example, can try again if the first bite does not succeed. In the most favourable case, an animal may have virtually unlimited opportunities to try to progress in the sequence, without losing what has been accomplished so far. Such a sequence could be learned rapidly (it takes *ml*(*l*+1)/2 time steps to learn the complete sequence, see appendix). We will study a concrete example of a favourable exit pattern when discussing primate stone tool use below.

### 3.3. Availability of information

In this section, we consider two further determinants of the efficiency of chaining, both concerning the information available to the animal. Indeed, these factors affect any learning mechanism, not only chaining. The first one is the extent to which stimuli are informative about environmental states. So far, we assumed that the animal can perceive unambiguously which environmental state it is in. In reality, environmental states have to be inferred from sensory information, which may contain redundant or uninformative stimuli, and not provide complete information. Additionally, environments often present animals with novel configurations of stimuli, some known and some unknown. Animals need thus to generalize knowledge from familiar situations to novel ones. These difficulties have been dealt with extensively in both the animal and the machine learning literature [41,72–75]. An extension of our model that operates based on sensory information is presented in the appendix, but is not discussed for brevity. A second informational dimension of environments is their predictability. Day–night transitions, for example, are more predictable than sun–rain transitions; hunting for a carnivore is typically less predictable than foraging for a herbivore. In general, learning is easier if environmental states follow one another consistently and if the consequences of behaviour are fixed rather than variable, because in these cases fewer experiences are necessary to estimate satisfactorily the value of states and actions.

In this paper, we do not deal extensively with these issues, although §(c) shows that lack of information can have remarkable effects. Clearly, lack of information and predictability can limit the attainment of optimal behaviour. At the same time, however, being able to perceive or gather more information is not necessarily advantageous because it increases learning costs. An animal with keener sense organs, for example, will be able to distinguish between more stimuli, which can be counterproductive if not supported by effective generalization. If every banana looks entirely unique, for example, one will have to learn the value of each one separately. A similar caveat applies to memory. In many instances, sequences of stimuli contain more information than just the last stimulus (e.g. a cheetah may do well to give up hunting a gazelle if it recently saw lions in the vicinity). Taking into account this information, however, may render learning unfeasible. Given that *n* stimuli can be perceived, remembering sequences of *a* stimuli leads to being able to distinguish between *n*^*a*^ sequences of stimuli, each with a potentially unique meaning. Remembering this information would carry both memory costs (more storage would be needed) and learning costs, because each sequence would be experienced fewer times, and the learner would have to generalize across sequences rather than stimuli. These costs are probably the reason why most animals have rather limited working memory [76], which is economical but may restrict their abilities to learn about environmental regularities across time spans longer than a few minutes. When longer-lasting memories are necessary, animals seem to have evolved specialized memory mechanisms, supported by genetic predispositions, rather than improving their general-purpose memory. For example, hoarding specialists such as Clark's nutcrackers can cache 30 000 seeds in a season and remember their location for months [77,78], but they cannot remember the colour of a light for more than about a minute [79]. We do not pursue this issue further, but we note that our chaining model could be applied to both specialized memories and general-purpose memories holding more than the last stimulus. For example, we could replace *w*_*S*_ with *w*_(*S*,*S*′)_, where (*S*,*S*′) is a sequence of two successive stimuli. In this way, sequences of two stimuli would acquire value, with all costs and benefits that this would entail. The model could also operate on episodic-like memories of the kind suggested in [80]. Likewise, there are many models of generalization that can be applied to the problem of learning from sensory information rather from direct information about environmental states (appendix; [41,72–74]).

## 4. Genetic control of learning and decision making

The chaining model contains parameters that can have profound effects on decision making and learning. These are *u*_*S*_, *β*, *α*_v_, *α*_*w*_ and the initial values for *v*_*S*→*B*_. In this section, we ask whether, by appropriately structuring these parameters, chaining can encompass not only learning phenomena, but also instinctual aspects of behaviour. The latter have traditionally been studied by ethologists, who were interested in species-specific behavioural adaptations and considered learning to be under tight genetic control [81,82]. Contemporary cognitive ethology also appears to view cognitive capacities (planning, episodic-like memory, ‘physical cognition’, etc.) as traits that can be either present or absent in a species, and therefore, under strong genetic control. Experimental psychologists, on the other hand, sought general principles of learning that would apply to all species [30,83]. These fields got slowly closer over time, and today many scholars recognize that similar learning principles operate across species, at the same time that genetic predispositions can tailor learning to each species' needs [17,34,84–86]. Indeed, without at least some genetic guidance, there would be too many possible action sequences for learning to succeed. Recall that an animal with a repertoire of *m* behaviours trying to learn a sequence of length *l* may have to try out ∼*m*^*l*^ sequences before finding the correct one. Given that the behaviour repertoire cannot be reduced below a certain size, and excepting favourable entry and exit patterns, it would seem that only short sequences (perhaps only single behaviours) could be learned [87]. Genetic predispositions offer a solution to this conundrum.

A first observation is that animals rarely use their full behavioural repertoire in any given context [17,84,85,88]. Squirrels, for example, cannot open nuts from birth, yet they are predisposed to use only a few behaviours while learning the correct technique, such as rotating the nut, biting and prying [89]. Genetic predispositions that limit the behavioural repertoire to relevant actions can be very effective [87]. For example, halving the number of behaviours that are tried reduces the number of possible sequences of length *l* to a fraction 1/2^*l*^ of the initial number (i.e. to about 13% with *l*=3 and to about 6% with *l*=4). In our model, predispositions to use certain behaviours in a given context can be implemented by letting *β* in equation (2.2) depend on external stimuli and motivational state (internal stimuli). When a hungry squirrel holds a nut, for example, the *β* value for prying, biting and rotating the nut could be increased so that only these behaviours are likely to occur.

Genetic control of *β* can also cause changes in exploration over the lifespan. If an animal's environment is stable, in fact, it may be profitable to explore a lot when young (low *β*) and to reduce exploration with age (increasing *β* with time, similar to simulated annealing in optimization [90]). In this way, a young animal can try out many different courses of action, and therefore, identify the best ones. An older individual, however, would restrict its behaviour to the best known options, devoting its time primarily to exploiting acquired knowledge.

Other genetic predispositions are manifest in what animals ‘pay attention to’. With the latter we mean that, when many stimuli are experienced jointly, animals may preferentially learn about some rather than others. For instance, when presented with a flavour+sound stimulus that predicts illness, rats associate only flavour with illness. If the same stimulus predicts shock, however, rats associate the sound with shock [91,92]. Similarly, hamsters that are reinforced with food learn readily to dig and rear (part of their natural foraging repertoire), but not to groom or nest-build [93,94]. The evolutionary rationale for these phenomena is that they encode stable properties of the organism–environment interaction, e.g. that illness is caused by food and not by sounds, or that digging and rearing are useful in procuring food, while grooming and nest building are not. These effects can arise in our model by letting *α*_v_ in equation (2.4) depend on *S* and *S*′. A high value results in easily established associations; a low or null value in associations that are hard or impossible to learn.

In general, any parameter of the chaining model can be made dependent on external and internal stimuli (motivational states, proprioception, etc.), which can generate predispositions to choose or to learn certain behaviours, and to seek certain outcomes (by appropriately structuring primary reinforcement, *u*_*S*_). The resulting genetic control can range from lax (most behaviours can be chosen and most associations learned) to strict (e.g. in some cases the repertoire could be limited to a single behaviour, or a limited set of associations could be formed). In this way, it is possible to unify the view that behaviour has instinctual components with the view that decision making and associative learning rely on general mechanisms that are functionally conserved across many taxa. There is thus no conflict between the ideas of general learning mechanisms and of evolution adapting learning and behaviour to a species' niche. In our model, the role of genetic evolution is to optimize a well-defined set of learning parameters to balance the costs and benefits of learning and behavioural flexibility. Table 2 suggests what phenomena can so arise, but it will take considerable research to evaluate the merits of this proposal. In §(b), we consider several examples of puzzling learning phenomena that receive a remarkably simple explanation when predispositions are taken into account.

**Table 2.** Possible influences on decision making and learning of the model parameters in equations (2.2), (2.4) and (2.5), when these are understood as depending, potentially, on external stimuli (*S*, *S*′), on internal stimuli (*X*, *X*′) and on the behaviour performed (*B*). The internal stimuli referred to by *X* and *X*′ are motivational states, internal clocks, hormonal states and so on. These are not discussed explicitly in the text, but here we emphasize that they can also influence decision making and learning. The notation in the table refers to the sequence (*S*,*X*)→*B*→(*S*′,*X*′) in which external stimulus *S* is experienced together with internal stimulus *X*, then behaviour *B* is chosen and then the external and internal stimuli *S*′ and *X*′ are experienced.

parameter	may depend on	influences	enables genes to control
*β*, response bias	*S*, *X*, *B*	selection of behaviour	amount of exploration
			response bias (different *β*s for different *B*)
			context dependence (different *β*s for different *S* or *X*)
*u*, primary reinforcement	*S*′, *X*′	rate of change and maximum values of *v* and *w*	what responses can be learned, and how fast
			what the animal ultimately strives for (what is a reinforcer)
*α*_v_, learning rate	*S*, *X*, *B*, *S*′, *X*′	rate of change of *v*	what responses can be learned (*α*_v_>0), and how fast
			context dependence (different *α*_v_s for different *S*, *X*, *B*, *S*′, *X*′)
*α*_*w*_, learning rate	*S*, *X*, *S*′, *X*′	rate of change of *w*	what can become a conditioned reinforcement (*α*_*w*_>0), and how fast
			context dependence (different *α*_v_s for different *S*, *X*, *B*, *S*′, *X*′)
			behaviour sequences

## 5. Applications to learning phenomena

In this section, we apply our chaining model to learning phenomena from studies in the laboratory and in the wild. Our goal is to show that chaining can reproduce a host of learning phenomena, even when these appear not to conform with current opinion on associative learning.

### 5.1. Chaining in nature

Natural resources can be exploited only through specific behaviour sequences. As discussed above, these sequences can be realistically learned through chaining only if they are relatively short, if favourable entry and exit patterns occur, or if the animal has some knowledge (innate or previously acquired) that guides it towards the correct sequence. Some resources appear relatively easy to exploit through chaining. Many animals, for example, rely on accessible resources such as fruit or other plant matter. A bird, for example, may need to learn a sequence such as:

Tree→Approach→Fruit→Peck→Openfruit→Lick→Sweet

where italic shape indicates stimuli and upright actions. The last stimulus, *Sweet*, represents the reward. Such a sequence would be easy to learn by chaining provided open fruits, fruits and trees are encountered often enough while exploring the environment (similarly to the random entry pattern considered above), and given that mistakes are not catastrophic. For example, many pecks can be attempted if the first one does not succeed. Of course, learning would be even easier with appropriate genetic predispositions, e.g. to peck objects with a certain smell, colour or size, to spend time in trees and so on. Reports that foraging efficiency often improves with age support the idea that foraging strategies are partly learned (e.g. [95–97]).

**Figure 4.**
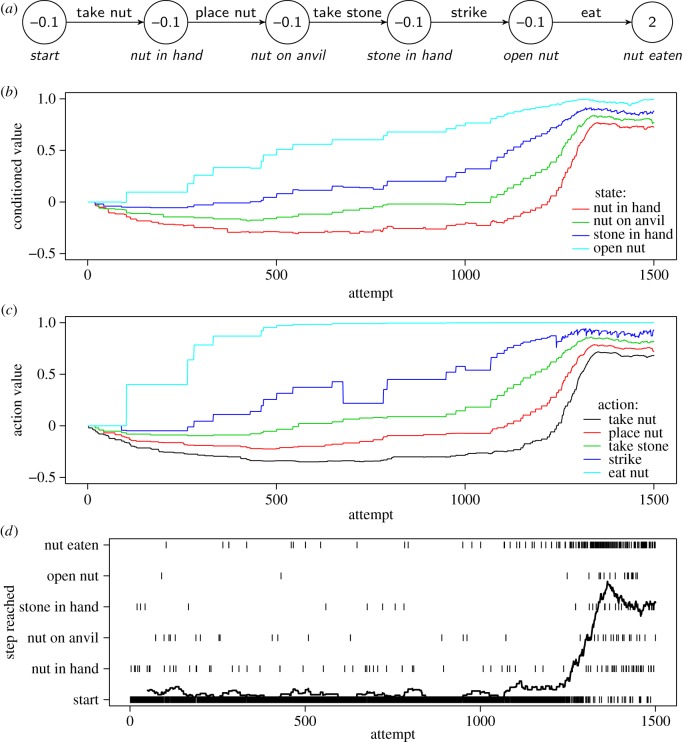
Model of nut cracking in chimpanzees, based on Inoue-Nakamura & Matsuzawa [98]. (*a*) Task model. Only correct actions are shown; incorrect actions lead back to the initial state with probability 0.25 and have no effect with probability 0.75. The probability that a strike results in opening a nut is 0.75, and when a strike fails the animal can try again. Lastly, there is a probability of 0.01 to advance from the first state directly to *Open nut*, corresponding to the young chimpanzee stealing an open nut from its mother. The three graphs show the progression of learning through chaining. (*b*,*c*) The conditioned values of states and the estimated values of state-behaviour pairs, respectively. Panel (*d*) records the highest state reached in each attempt; the solid line is a smoothed running average. Model parameters where *α*_v_=0.05, *α*_*w*_=0.05 and *β*=5. Starting *v*_*S*→*B*_ values were set so that the five functional behaviours were selected with a probability of ≃2.5% each, consistent with selecting behaviours randomly from a repertoire of about 40.

Can chaining also learn to exploit more complex resources? By way of example, we consider the use of tools to crack nuts by some groups of chimpanzees (*Pan troglodytes*) and capuchin monkeys (*Cebus apella*), which is one of the most remarkable cases of sequence learning in the wild [98,99]. Our goal is to show that, while nut cracking is undoubtedly a complex skill, an analysis of entry and exit patterns reveals a number of favourable circumstances that enable chaining to learn a complete nut-cracking sequence in a realistic number of attempts. We base our model on the detailed observations by Inoue-Nakamura & Matsuzawa [98] of chimpanzees at Bossou, Guinea. These authors have described nut cracking as a sequence of five behaviours: picking up a nut, placing it on a stone used as anvil, picking up a stone used as hammer, striking the nut with the hammer and finally eating the nut (figure 4*a*). Chimpanzees manipulate stones and nuts from around six months of age, but they do not become proficient nut openers until 3.5–5 years of age. This learning may seem slow, until one realizes the complexity of the task. Chimpanzees do not seem to have specific predispositions for this task, and appear aided only by a general propensity to manipulate objects. Indeed, Inoue-Nakamura and Matsuzawa observed naïve chimpanzees perform approximately 40 behaviours directed at nuts and stones, including many non-functional ones such as throwing nuts to the ground, kicking stones and attempting to eat unopened nuts. Thus, the young chimpanzee must locate a functional sequence among ≃40^5^≃100 million possible ones. Even if there were hundreds or thousands of functional sequences, the task would be practically impossible. However, several facilitating circumstances apply. First, the young has plenty of time to learn, with both stones and nuts readily available for years. Additionally, mistakes have rather mild consequences. For example, if a nut rolls away or is placed on the ground rather than on the stone anvil, corrective action can often be taken without starting from scratch. Lastly, young chimpanzees occasionally steal an open nut from their mother. This establishes the open nut as a conditioned reinforcer and effectively shortens the sequence to be learned by one step. We have included these features in the model in figure 4 as follows:

— A transition from *Start* to *Open nut* is possible, directly, with probability 0.01.
— Mistakes leave the animal in the same state with probability 0.75; with probability 0.25 the animal goes back to *Start*.
— A strike succeeds with probability 0.75, so that more than one strike is often necessary.

The graphs in figure 4 show a simulation in which the chaining model becomes proficient at nut cracking in about 1500 attempts (across simulations, both longer and shorter times occur; the time is also influenced by various parameters such as *α*_v_ and *α*_*w*_). The top graph refers to the conditioned values of states. As expected, states that are more proximal to the reward acquire value before more distal states. Note that state *Open nut* acquires value early on due to the simulated animal stumbling upon a few open nuts, and then choosing behaviour Eat, initially by chance. Note also that the value of most states initially decreases: having a nut is worth nothing unless one can open it (cf. our self-control example above). The middle graph shows the value of the correct action in each state. In the lower graph, the ticks indicate which point in the sequence is reached in each attempt. The line is a running average over 50 attempts. A remarkable feature of this graph is that nut cracking seems to appear relatively suddenly over the course of about 100 attempts. This happens because complete sequences require, by definition, all steps to be learned. As the correct behaviours begin being chosen, more and more rewards are collected, which further strengthens the correct behavioural tendencies in a positive feedback cycle, which results in the impression of relatively sudden learning. In reality, we can see that the values of correct actions had been growing gradually all along, supported by conditioned value accruing to the intermediate states.

While our model of nut cracking may be simplified (it neglects, for example, that motor learning also needs to take place), it still requires the learner to locate a single sequence out of approximately 100 million. That this can be accomplished in about 1500 attempts (about 4 years with just one attempt per day) demonstrates that complex behavioural strategies can be learned through chaining. Note that we did not assume any specialization for social learning (e.g. imitation or emulation abilities), which is often believed to be necessary to acquire nut cracking. According to our model, the main role of the young's social environment may be to provide enhanced opportunities for learning [100,101]. In this view, complex tool use is rare not because it requires specific cognitive mechanisms for, e.g. social learning or causal reasoning. Rather, tool use may be rare simply because it requires learning longer sequences, and because entry patterns may be much less favourable before tool use is established. For example, the first chimpanzees that pioneered nut cracking may not have had easy access to open nuts and suitable stones.

Similar considerations apply to nut-cracking behaviour in capuchin monkeys and to other well-known cases of sequence learning in the wild. For example, black rats (*Rattus rattus*) can learn to feed on pine seeds [102]. The rats first obtain a closed pine cone, then hold it firmly to bend and then detach a cone scale with teeth, and finally eat the seed thus uncovered. The sequence is then repeated until all seeds have been extracted. Aisner & Terkel [103] manipulated experimentally the entry pattern to this sequence. The rats could not learn the sequence, even after three months, unless they entered it near the rewarding state, by being provided with partially opened cones with exposed seeds. Even in this case, rats sometimes failed to learn the whole sequence. Only young rats raised by proficient mothers (including foster mothers, to control for genetic effects) consistently learned the whole sequence. Aisner and Terkel observed that pups gathered around the mother while she was extracting seeds, and sometimes could obtain and eat a seed. Older pups stole partially opened cones from the mother, carrying them to safety and trying to extract the seeds. This pattern of acquisition, starting from eating seeds, then handling partially opened cones, and finally learning the complete sequence, is clearly suggestive of chaining.

### 5.2. Misbehaviour and auto-shaping

To illustrate how genetic control can influence decision making and exploration we apply our model to the phenomena of misbehaviour, in the sense of Breland & Breland [104] and auto-shaping [105]. In these phenomena, a response develops in the course of learning that is unnecessary or counterproductive [17,106,107]. A paradigmatic example reported by the Brelands concerns training a raccoon to deposit a token into a machine to get a food reward. Progress on this task was initially rapid, but over time the animal started to hold on to the token, eventually depositing it only after much hesitation. A similar example is wheel running in rats: in a situation in which both food and a running wheel are available, rats can develop a tendency to run on the wheel prior to eating, thus expending energy unnecessarily [108]. Lastly, in the original auto-shaping study [105], pigeons spontaneously developed pecking a light that was turned on just before food delivery, despite this behaviour being unnecessary to obtain food.

**Figure 5.**
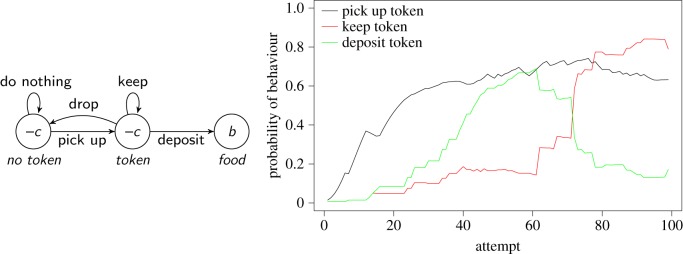
Simulation of ‘misbehaviour’ due to innate bias in choice of behaviour [104]. Left: simple model of a raccoon being taught to pick up a token and deposit it in a ‘bank’ in exchange for food. To simulate misbehaviour, we set *β*=1 for all state-behaviour pairs, apart from *β*=3 for action ‘keep’ in state ‘token’, creating a bias for holding onto the token as the latter acquires conditioned value. We also set *v*_no token→do nothing_=*v*_token→drop_=5 initially to model the fact that the correct actions are chosen only rarely at first; other *v* values start from zero. Other parameters are *c*=0.2, *b*=2, and *α*_v_=*α*_*w*_=0.25. Right: results from a representative simulation, showing that the chaining model first learns the correct actions (pick up the token, then deposit it), but later switches to holding on to the token. See text for analysis.

In these examples, we expect the token, the running wheel and the light to acquire conditioned value because their presence predicts a reward, but this is not sufficient to explain why sub-optimal actions are directed at them. The latter is easily accommodated, however, by letting *β* values in equation (2.2) depend on stimuli and behaviours, thereby creating predispositions for certain behaviours in certain states. Figure 5 shows a model of the above-mentioned raccoon example in which we used a higher *β* for keeping the token. As in the Brelands' report, the model initially progresses rapidly, learning to pick up and deposit the token, but after a while it starts to keep the token, and eventually deposits it only with low probability. The reason that misbehaviour is not immediately apparent is that the higher *β* value has no effect unless *v*_token→keep_ is positive, and *v*_token→keep_ cannot become positive until the token has acquired conditioned value (*w*_token_>0).

**Figure 6.**
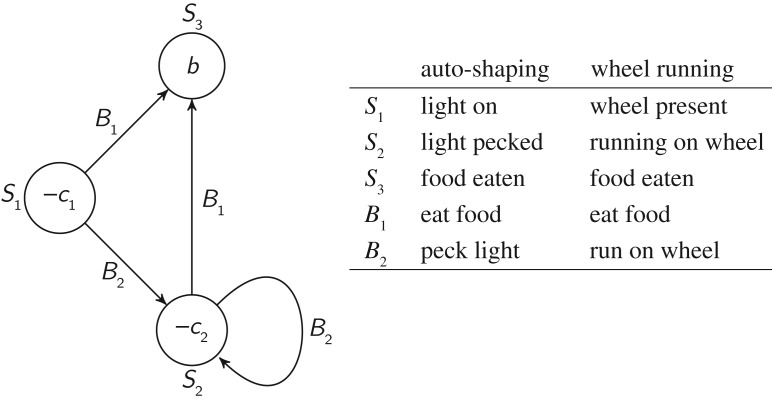
A generic model of misbehaviour that can be applied to various phenomena, such as auto-shaping [105] and schedule-induced wheel running [88]. An animal is assumed to have two behavioural options. ‘Useful’ behaviour (*B*_1_) leads to a reward, ‘useless’ behaviour (*B*_2_) leads to a second state in which the same behavioural options are available. In other words, every time *B*_2_ is chosen, a cost −*c*_2_ is incurred, without any influence on the possibility of reaching the reward.

Similar set-ups can reproduce wheel running, auto-shaping and other misbehaviour phenomena. Figure 6 shows a generic model of misbehaviour in which the animal can choose between a useful behaviour (*B*_1_) and a useless or costly one (*B*_2_). The latter is assumed to have a higher *β* value in state *S*_1_, to model the emergence of misbehaviour, and also possibly in *S*_2_, to model the fact that misbehaviour may interfere with completing the sequence. The figure also details how the model applies to auto-shaping and wheel running. Although several adjustments would be necessary to replicate experimental conditions precisely, the model makes the general point that an unproductive behaviour can be favoured because of a genetic predisposition to express the behaviour in response to stimuli that have acquired conditioned value.

Technically, the reason why chaining can cause misbehaviour in the cases treated above is that the decision function does not honour the ranking of state-behaviour values, leading to actions with lower estimated value being chosen more often than actions with higher values. This violates the conditions under which chaining can converge to optimal behaviour (see appendix), and it may appear puzzling that the optimization abilities of chaining may have been so spoiled. The predispositions that result from manipulating *β* values, however, can be beneficial if they bias behaviour toward what is usually profitable in the animal's natural environment: it is good for raccoons to hold onto food, for rats to move about to find food and for pigeons to peck at food items. Computationally, these predispositions can be understood as a trade-off between being able to learn optimal behaviour in any environment, but possibly slowly, and being able to learn quickly, but only in environments in which the predispositions are appropriate.

We conclude this section with a novel insight into the nature of predispositions afforded by the chaining model. Sometimes genetically predisposed behaviour is readily apparent. For example, toads spontaneously attack moving objects with the characteristics of prey [109]. In our model, these responses correspond to initially high *v*_*S*→*B*_ values. In the cases discussed here, however, predispositions emerge during the course of learning, which we have modelled with a higher *β* value. This indicates that, in nature, the predisposition has evolved to act on stimuli whose recognition is *learned,* in which case a high *v*_*S*→*B*_ cannot evolve because the stimulus *S* is unknown. This is testament to the crucial role of learning in natural behaviour and is coherent with a wealth of results showing that even stimuli proximal to primary reinforcements are often not recognized innately. For example, dogs do not salivate at the sight of meat until they have tasted it a few times [110,111], and similar results have been obtained with many mammals and birds [5,6].

### 5.3. A case of missing information

An experiment by Cronin [112] illustrates further how chaining can illuminate apparently puzzling behaviour, in this case arising from the animal lacking information about environmental states. In the experiment, pigeons could peck either of two stimuli, *S*_red_ and *S*_green_ (lights of different colours), simultaneously present. Pecks to *S*_red_ resulted in access to food; pecks to *S*_green_ did not. Ordinarily, this would be an easy discrimination, but in Cronin's experiment the reward was delayed. That is, all pecks caused *S*_red_ and *S*_green_ to turn off and a white light (*S*_white_) to turn on for 60 s, after which the pigeons could observe the consequences of their choice. Thus, the stimulus immediately preceding the reward was actually non-diagnostic, while the truly predictive stimulus was 60 s in the past. Under these conditions, pigeons failed to solve the discrimination and pecked red and green equally often. According to the chaining model, *S*_white_ acquired conditioned value because it was followed by a reward, half of the time. The conditioned value of *S*_white_ then reinforced pecking at both *S*_red_ and *S*_green_ equally. Cronin further investigated how changes in the stimuli that intervene between *S*_green_ and *S*_red_ and the reward would influence the pigeons. For a second group of pigeons, pecks to *S*_red_ caused a yellow light (*S*_yellow_) to appear during the delay, while pecks to *S*_green_ caused a blue light (*S*_blue_). These pigeons could solve the task, eventually pecking red about 95% of the time. In this case, *S*_yellow_ is followed by food while *S*_blue_ is not. Consequently, only *w*_*S*_yellow__ grows, and can reinforce pecking *S*_red_ selectively.

**Figure 7.**
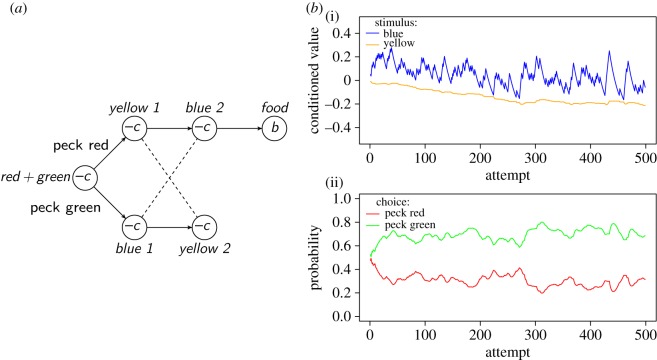
(*a*) Diagram of the task faced by pigeons in the ‘reverse-cue’ group in [112]. In the initial state, a red and a green light were present. If pigeons pecked red, they saw a sequence of a yellow light followed by a blue light, followed by food. If they pecked green, they saw a blue light first, followed by a yellow light and no food. Because of working memory limitations, pigeons could not distinguish between the states indicated by lights of the same colour (represented by dashed lines). The parameter −*c* is the cost of being in non-rewarding states (*c*=0.2 in our simulation), the parameter *b* the value of the food reward (*b*=1). (*b*) Simulated behaviour of the chaining model on the task. (i) Conditioned value of the yellow and blue lights. The blue light has more variable conditioned value because of higher variance in the value of the following state. (ii) Probability of pecking the red or the green light. Parameter values: *α*_v_=0.1, *α*_*w*_=0.05 and *β*=5.

Cronin's most intriguing result was obtained with a third group of pigeons. For these pigeons, pecks to red caused the stimulus sequence *S*_yellow_→*S*_blue_→*S*_food_, while pecks to green caused the sequence *S*_blue_→*S*_yellow_→*S*_no food_ (figure 7*a*). The chaining model predicts that *S*_blue_ should acquire conditioned value, which would reinforce pecks to the *incorrect* stimulus, *S*_green_, because pecking *S*_green_ would produce the now valuable *S*_blue_ (figure 7*b*). This is exactly what Cronin observed: pigeons in this group chose green about 90% of the time. We have successfully simulated this result (figure 7*b*) assuming that pigeons cannot distinguish between the states *Blue1* and *Blue2*, nor between *Yellow1* and *Yellow2*. This inability derives from the same working-memory limitations apparent in the first experimental group [76]. Thus, from the point of view of the animal, *S*_yellow_ identifies a state that is followed half of the time by *S*_blue_ and half of the time by *S*_no food_. Neither of these are rewarding. *S*_blue_, on the other hand, is followed half of the time by the non-rewarding *S*_yellow_ and half of the time by the rewarding *S*_food_. Thus *S*_blue_ acquires conditioned value. One might expect this conditioned value to spread to *S*_yellow_ to some extent, because *S*_yellow_ sometimes precedes *S*_blue_. However, as *S*_blue_ acquires value, the model starts to choose Peck Green and, therefore, most of the time *S*_yellow_ precedes the start of the next trial rather than *S*_blue_.

In summary, chaining explains readily Cronin's puzzling results—as suggested by Cronin herself [112]. Chaining can optimize behaviour when information is sufficient, but it can also learn grossly sub-optimal behaviour when information is lacking.

## 6. Discussion

We have introduced a model of ‘chaining’—associative learning with conditioned reinforcement—and used it to explore the role of learning in the ontogeny of optimal behaviour, as well as the relationship between learned and instinctual aspects of behaviour. According to our analysis, the model has many advantages:

— *Simplicity*: Chaining is completely described by three, computationally simple equations. Decision making rests solely on the comparison of state-behaviour values, equation (2.2), while both learning equations use only immediately available information, equations (2.4) and (2.5).
— *Power*: Chaining can optimize behaviour sequences, and thereby reconcile observations that behaviour is optimal or near-optimal with the fact that behavioural strategies are often shaped by learning, and therefore, cannot be optimized fully by genetic evolution.
— *Integration with genetic information*: Chaining is not only a model of learning: it also naturally accommodates genetic influences on learning and behaviour. While chaining has *a priori* unlimited flexibility, what is actually learned can be brought under genetic control by evolution tuning model parameters. Such tuning can result in predispositions that lead individuals to develop species-specific behaviour, such as exploiting resources that define the ecological niche of the species [81,85]. Therefore, chaining can account for the species-specificity of behaviour that initially led ethologists to postulate tight genetic control of behaviour.
— *Evolutionary plausibility*: Simplicity, power and the possibility to control learning genetically lend strong evolutionary plausibility to chaining. Chaining can evolve from simpler associative learning through the sole addition of conditioned reinforcement. This evolutionary step transforms a mechanism capable of learning only stimulus–response relationships into one that can learn arbitrary sequences of behaviour. Furthermore, evolution can tune learning parameters to re-adapt learning following environmental changes.
— *Empirical support*: Conditioned reinforcement, the key learning process in chaining, is solidly established in birds and mammals [27,113,114]. Moreover, animal trainers have used techniques based on conditioned reinforcement with well over 100 species (Bob Bailey, personal communication, and [31]), see http://www3.uca.edu/iqzoo/iqzoo.htm, for examples. This widespread occurrence indicates that, in all likelihood, conditioned reinforcement serves an important role in natural behaviour.
— *Misbehaviour*: Chaining can also model how animals sometimes learn sub-optimal strategies. Sub-optimal behaviour is a valuable test of behaviour models. If behaviour were invariably optimal, its underlying mechanisms would be obscured, and all models capable of producing optimality would receive equal support. Sub-optimal behaviour, however, depends strongly on underlying mechanisms: while there is, typically, only one way to be optimal, there are many possible kinds of mistakes. Therefore, predicting mistakes is more challenging than predicting optimal behaviour. We regard the preliminary successes reported in §§(b) and (c) as a strong indication that chaining captures fundamental aspects of learning, including its interaction with genetic predispositions.

Based on these results, we believe that the formulation of more complete associative learning models, as attempted here, can clarify the role of learning in behavioural evolution and help resolve long-standing issues at the intersection of ethology (the nature of instinct, constraints on learning), psychology (sequence learning, misbehaviour), behavioural ecology (optimization) and animal cognition (what mental capacities animals possess). Of relevance to the last field, our chaining model clarifies what associative learning can accomplish, and therefore, provides a benchmark against which other (cognitive) mechanisms can be evaluated. We already pointed out, for example, that chaining can learn self-control and that it forms expectations. Another example concerns the claim that animals are ‘stuck in time’, i.e. they react only to present needs and current stimuli, often referred to as the Bischof–Köhler hypothesis [115,116]. One consequence of this hypothesis is that animals would be unable to plan for the future. Observations and experiments that appear to contradict this claim (e.g. [117,118]) tempt researchers to hypothesize sophisticated mechanisms for planning and ‘mental time travel’, and to discard associative learning as a potential explanation. This conclusion, however, derives from equating associative learning with simple stimulus–response learning. As we saw in our self-control example, chaining can learn effective courses of actions that take into account the future. In that example, the sequence leading to the larger reward can be made arbitrarily long, provided the reward is worth working for (or waiting for). Indeed, chaining is currently the only formal model of animal learning that makes specific predictions on how plans may be learned: by back-tracking conditioned values in the presence of favourable entry and exit patterns. These predictions can be tested in experiments, but mere observations of planning are not informative about the mechanisms that generate plans.

We have deliberately left out important issues, partly because of space limitations and partly because of their complexity. For example, the mechanisms whereby animals learn the values of actions and of states are more nuanced than our model assumes, and indeed are not completely known [16,17]. At the same time, our model captures the fundamental fact that conditioned reinforcement enables animals to learn sequences of behaviours. We have also not discussed how animals infer environmental states from sensory information, and how they relate novel sensory inputs to familiar ones. The theory of these phenomena, however, is reasonably well developed [74,119]; a chaining model that operates based on sensory information is presented in the appendix. Lastly, we did not cover many learning scenarios relevant to natural environments. Social learning, for instance, was discussed only briefly in §(a). In future work, more attention should be paid to social stimuli and how they are processed. For example, one may explore the hypothesis that specific predispositions to behave in certain ways in the presence of social stimuli may lead to imitation at the behavioural level, in the absence of a specific cognitive capacity for imitation.

In conclusion, the combination of chaining and genetic predispositions results in powerful behaviour systems that can optimize behaviour even in the absence of ‘cognitive’ information processing. Moreover, the combination of chaining and genetic predispositions resolves naturally the debate over the concept of instinct (e.g. [120–122]). By introducing genetic control of learning parameters, we can recognize the existence of both general learning processes and of genetic information that, in natural environments, results in well-adapted, species-typical behaviour. We hope that this perspective can serve as a foundation for a unified theory of behaviour.

## Data accessibility

We programmed equations (2.2), (2.4) and (2.5) in the R language [123], together with a simple simulation framework to specify tasks and simulate the learning process. Our code is publicly available at figshare (https://dx.doi.org/10.6084/m9.figshare.3848199.v1). Source code and documentation are bundled as an Emacs org-mode file [124,125]. A PDF file documenting both code and simulations, and an R file containing code only are also provided.

## Appendix A. Model analysis

**A.1. Convergence**

Convergence of chaining to optimal behaviour can be analysed similarly to other reinforcement learning models [42,126]. Convergence in expectation can be established as follows. Let us start from the convergence of *w*_*S*_ values, and let **w** be the vector of such values. We introduce the operator *A*_*w*_ as the transformation of **w** into its expected next value:

A 1 $$A_{w}\text{w}=(1-\alpha_{w})\text{w}+\alpha_{w}\text{P}(\text{u}+\text{w}),$$

where **u** is the vector of *u*_*S*_ values, and **P** is the matrix of probabilities such that *P*_*S*,*S*′_ is the probability to go from *S* to *S*′ according to a given behavioural strategy that we assume is being followed (thus, **P** takes into account both environmental dynamics and behavioural choices). The expected course of learning consists of repeatedly applying *A*_*w*_ to successive values of **w**. Convergence of this process can be proved using Banach's fixed point theorem [127], i.e. establishing that, for any two vectors **w**^(1)^ and **w**^(2)^, we have:

A 2 $$∥A_{w}{\text{w}}^{\left(1\right)}-A_{w}{\text{w}}^{\left(2\right)}∥\leq a∥{\text{w}}^{\left(1\right)}-{\text{w}}^{\left(2\right)}∥,$$

where ∥⋅∥ is the Euclidean norm and 0≤*a*<1. equation (A 2) means that the distance between **w**^(1)^ and **w**^(2)^ decreases with each application of *A*_*w*_, and eventually vanishes. Because **w**^(1)^ and **w**^(2)^ are arbitrary, equation (A 2) implies that all vectors converge to the same fixed point, which can be verified to be the correct vector of state values given in equation (2.7), for which $A_{w}{\text{w}}^{*}={\text{w}}^{*}$. To establish equation (A 2) we reason as follows. First, we introduce the abbreviation

A 3 $$\text{d}={\text{w}}^{\left(1\right)}-{\text{w}}^{\left(2\right)}.$$

We then use equation (A 1) to write:

A 4 $$∥A_{w}\text{w}{}^{\left(1\right)}-A{\text{w}}^{\left(2\right)}∥=∥(1-\alpha_{w})\text{d}+\alpha_{w}\text{Pd}∥\leq (1-\alpha_{w})∥\text{d}∥+\alpha_{w}∥\text{Pd}∥,$$

where we have used Cauchy's inequality, ∥**x**+**y**∥≤∥**x**∥+∥**y**∥. Now we note that the Markov process of state transitions has a unique stationary distribution for the tasks we are considering, corresponding to the terminal state having been reached (what follows can also be adapted to the case of multiple terminal states). Hence **P** has a unique eigenvalue with value 1, and we can write

A 5 $$\text{P}={\text{pp}}^{t}+\text{Q,}$$

where **p** is the vector describing the stationary distribution, ^*t*^ denotes transposition and **Q** describes the rest of the process. Crucially, the eigenvalues of **Q** have magnitude <1, because otherwise there would be other stationary distributions. Notice now that on the terminal state we have $w_{S}^{\left(1\right)}=w_{S}^{\left(2\right)}=0$, because the terminal state has no successor and thus its value is never updated. This implies

A 6 $$\text{p}{}^{t}\text{d}=0.$$

Using equation (A 5) and equation (A 6), we get:

A 7 $$∥\text{Pd}∥=∥{\text{pp}}^{t}\text{d}+\text{Qd}∥=∥\text{Qd}∥<q∥\text{d}∥,$$

where *q*<1 is the magnitude of the largest eigenvalue of **Q**. Using equation (A 7) in equation (A 4), we finally prove equation (A 2) with *a*=1−(1−*q*)*α*_*w*_:

A 8 $$\begin{array}{rl} ∥A_{w}{\text{w}}^{\left(1\right)}-A_{w}{\text{w}}^{\left(2\right)}∥ & \leq (1-\alpha_{w})∥\text{d}∥+\alpha_{w}∥\text{Pd}∥ \end{array}$$

A 9 $$\begin{array}{rl} & \leq (1-\alpha_{w})∥\text{d}∥+\alpha_{w}q∥\text{d}∥ \end{array}$$

A 10 $$\begin{array}{rl} & =[1-(1-q)\alpha_{w}]∥\text{d}∥. \end{array}$$

We have thus established that repeatedly updating a conditioned value vector **w** according to equation (A 1) leads to convergence to the correct **w*** for a given behavioural policy, i.e. in our model, for a given set of *v*_*S*→*B*_ values. In our model, however, updates to **w** do not proceed quite according to equation (A 1), which prescribes updating the whole **w** at once according to the average consequences of decisions. Rather, equation (2.5) updates one *w*_*S*_ at a time based on the consequences of each decision. Work in reinforcement learning shows that also this update rule converges to the correct **w*** values, subject to some assumptions [126], Proposition 4.4, p. 156.

Once the *w*_*S*_ values have converged, updating *v*_*S*→*B*_ according to equation (2.4) leads to *v*_*S*→*B*_ values that are closer to the *u*_*S*_+*w*_*S*_ values. Hence actions with higher *w*_*S*_ values will be chosen more often and performance will improve. (The latter statement is a slight extension of the policy improvement theorem [42], based on the fact that, while the updated *v*_*S*→*B*_ will not choose the best action exclusively, as assumed in the standard version of the theorem, they still lead to the best action being chosen more often.) Once both *w*_*S*_ and *v*_*S*→*B*_ values have stabilized, we can repeat the cycle and obtain further improved values of *w*_*S*_ and *v*_*S*→*B*_, and so on until an optimum is reached.

Updating *w*_*S*_ and *v*_*S*→*B*_ values alternatively, as just described, is a form of ‘policy iteration’ [41,126]. Animals, however, appear to learn conditioned values and responses in parallel. In our simulations, we always used equations (2.4) and (2.5) simultaneously, after each state transition. This is an example of ‘approximate optimistic policy iteration’ [126]. While approximate optimistic policy iteration is not guaranteed to converge in general, many such algorithms work well in practice [126,128,129]. The specific conditions under which our chaining model converges need yet to be evaluated, which is the topic of ongoing research.

**A.2. Learning times**

To estimate the learning times in figure 3 and table 1, we consider the task of learning a sequence of *l* behaviours in the following task:

— An attempt starts from state −1. From here, the animal either goes to any state between 0 and *l*−1 with probability *p*/*l*, or it goes to state 0 with probability 1−*p* (the total probability of going to state 0 is *p*/*l*+1−*p*).
— In states between 0 and *l*−1, one behaviour brings the animal to the next state; all other behaviours bring it back to state −1.
— In state *l*, the animal collects a reward and goes back to state −1.

This task models the entry patterns discussed in §(b). We assume that the animal always performs the correct behaviour if it has been learned; otherwise, it chooses a behaviour at random. With *m* behaviours, this results in a probability of *q*=1/*m* to choose the correct behaviour by chance. This behaviour approximates that generated by equation (2.2) in the limit of large *β* and no prior knowledge about correct behaviour.

To further simplify matters, we assume that each step is either ‘learned’ or ‘not learned’. To learn a step, the animal has to experience a rewarded transition to the next step, and this has to occur *a* times. The reward experienced can be primary or conditioned. In other words, we are approximating the sigmoid learning curves typical of our model with a step function. Note that there is no learning at all until the reward has been reached *a* times. When this happens, the last step is learned, and the learner is faced with an identical problem, just one step shorter. Thus, if *t*_*i*_ is the time to learn the last step of an *i*-step sequence, the time to learn a whole sequence of length *l* is $\sum_{i=1}^{l}t_{i}$.

It is possible to calculate *t*_*i*_ directly but it is convenient to split the calculation into two steps. We first calculate the number of *attempts* required to learn the last step of a chain of length *i*, written *n*_*i*_. We then calculate how long each attempt lasts, say *s*_*i*_. Because all attempts are statistically identical (recall that there is no learning until the reward is reached *a* times), we have *t*_*i*_=*n*_*i*_(*s*_*i*_+*l*−*i*). The *l*−*i* term takes into account the time taken to perform the known steps of the sequence. To calculate *n*_*i*_, we have to calculate how many times the animal goes through state −1 before reaching state *l* from the first time, when starting from state −1. This number, multiplied by *a*, is *n*_*i*_.

Let *b*_*k*_ be the expected number of visits to state −1 before reaching state *l* for the first time, and starting from state *k*. Our goal is to calculate *b*_−1_. To this effect, we recall that the expected value of a scalar quantity with finite expectation that is incremented or decremented on each state of a Markov chain can be calculated from a linear system of the form:

A 11 $$b_{k}=x_{k}+\sum_{j}p_{kj}b_{j},$$

where *b*_*k*_ is the expected value of the variable when starting from state *k*, *x*_*k*_ is the increment to the variable when going through state *k*, and *p*_*kj*_ is the transition probability from *k* to *j*. Equation (A 11) is a direct consequence of the Markov property: if we start in state *k*, the variable accrues an increment *x*_*k*_ and then goes to the next state *j*, from which the expected value is *b*_*j*_. Because the process is in general stochastic, we average with respect to the next state *j*. In the present case, the variable of concern is the number of passages through state −1, which is incremented by one only when we pass through this state. Hence we have *x*_−1_=1 and *x*_*k*_=0 for *k*≠1. Using the transition probabilities introduced above, equation (A 11) becomes:

A 12 $$\begin{array}{rl} b_{-1} & =1+(1-p)u_{0}+\frac{p}{l}\sum_{k=0}^{l-1}b_{k},\\ b_{k} & =(1-q)b_{-1}+qb_{k+1}\;0\leq k\leq l-1\\ \;\mathrm{and}\;b_{l} & =0, \end{array}\}$$

where the last equations takes into account that we are only concerned with the process until state *l* is reached. Now, let (1−*q*)*b*_−1_=*c*. Going backwards from the last equation in equation (A 12) we obtain:

A 13 $$\begin{array}{rl} b_{l-1} & =c, \end{array}$$

A 14 $$\begin{array}{rl} b_{l-2} & =c+qc \end{array}$$

A 15 $$\begin{array}{rl} \;\mathrm{and}\;b_{l-3} & =c+q(c+qc)=c+qc+cq^{2} \end{array}$$

and, in general,

A 16 $$b_{l-k}=c\sum_{i=0}^{k-1}q^{i}=c\frac{1-q^{k}}{1-q},$$

which can also be written

A 17 $$b_{k}=(1+b_{-1})(1-q^{l-k}).$$

Substituting this value in the first equation in equation (A 12) we have

A 18 $$b_{-1}=1+(1-p)b_{-1}(1-q^{l})+\frac{p}{l}b_{-1}\left(l-q^{l}\sum_{k=0}^{l-1}q^{-k}\right),$$

which can be solved for *b*_−1_ as follows. We have

A 19 $$q^{l}\sum_{k=0}^{l-1}q^{-k}=q\frac{1-q^{l}}{1-q}$$

and so

$$\begin{array}{rl} b_{-1} & =1+b_{-1}(1-(1-p)q^{l}-\frac{p}{l}q\frac{1-q^{l}}{1-q})\\ & =1+b_{-1}d_{l}(p,q), \end{array}$$

where we have defined the parenthesis that multiplies *b*_−1_ on the r.h.s. as *d*_*l*_(*p*,*q*) for brevity. We have finally obtained:

A 20 $$b_{-1}=\frac{1}{1-d_{l}(p,q)}$$

as expected the number of attempts until state *l* is reached for the first time given random behaviour. The values for the cases *p*=0 (entry from the start) and *p*=1 (random entry) are:

$$\begin{array}{rl} d_{l}(0,q) & =1-q^{l}\\ b_{-1} & =\frac{1}{q^{l}}=m^{l} \end{array}$$

and

$$\begin{array}{rl} d_{l}(1,q) & =1-\frac{q}{l}\frac{1-q^{l}}{1-q}\\ b_{-1} & =\frac{l}{q}\frac{1-q}{1-q^{l}}=(m-1)l\frac{m^{l}}{m^{l}-1}. \end{array}$$

Now let *s*_*k*_ be the expected time spent before returning to state −1, when starting from state *k*. Again, we are interested in calculating *s*_−1_. Time increments by 1 on every state, hence *x*_*k*_=1 for all *k*; equation (A 11) becomes

A 21 $$\begin{array}{rl} s_{-1} & =1+(1-p)s_{0}+\frac{p}{l}\sum_{k=0}^{l-1}s_{k},\\ s_{k} & =1+qs_{k+1}\;0\leq i\leq l-1\\ \;\mathrm{and}\;s_{l} & =1, \end{array}\}$$

where the last equation takes into account that from *l* return to −1 is certain, at which point the process stops. Backtracking as in the previous case we get

A 22 $$s_{k}=\frac{1-q^{l-k+1}}{1-q}.$$

We first calculate

A 23 $$\sum_{k=0}^{l-1}s_{k}=\frac{l}{1-q}-\frac{q^{2}(1-q^{l})}{{\left(1-q\right)}^{2}},$$

so that the first equation in equation (A 21) yields

A 24 $$s_{-1}=1+(1-p)\frac{1-q^{l+1}}{1-q}+\frac{p}{l}\left[\frac{l}{1-q}-\frac{q^{2}(1-q^{l})}{{\left(1-q\right)}^{2}}\right].$$

For *p*=0 and *p*=1, we have, respectively,

A 25 $$s_{-1}=1+\frac{1-q^{l+1}}{1-q}$$

and

A 26 $$s_{-1}=1+\frac{1}{1-q}-\frac{1}{l}\frac{q^{2}(1-q^{l})}{{\left(1-q\right)}^{2}}.$$

Note *s*_−1_ does not vary greatly with *p*, owing to the fact that in any case after at most a few moves one is back to state −1. In fact, for *l*=1, we have

A 27 $$s_{-1}=1+(1-p)(1+q)+p\left(\frac{1}{1-q}-\frac{q^{2}}{1-q}\right)=2+q$$

while for l→∞ we have

A 28 $$\lim_{l\to \infty }s_{-1}=1+(1-p)\frac{1}{1-q}+\frac{p}{1-q}\to 1+\frac{1}{1-q}=\frac{2-q}{1-q}.$$

For $q=\frac{1}{2}$, we have *s*_−1_|_*l*=1,*q*=1/2_=2.5 and $s_{-1}{|}_{l=\infty ,q=1/2}=3$. For $q=\frac{1}{10}$: *s*_−1_|_*l*=1,*q*=1/10_=2.1 and $s_{-1}{|}_{l=\infty ,q=1/10}=1.9/.9∼2.111\ldots$ . Therefore, *s*_−1_ is fairly uninteresting and we can focus on the number of attempts *b*_−1_.

Equation (A 20) gives us the expected time to learn one step of the chain. To learn all *l* steps we have to sum all learning times. For example, in the case of *p*=0 we have

A 29 $$\sum_{k=1}^{l}m^{k}=\sum_{k=0}^{l}m^{k}-1=\frac{1-m^{l+1}}{1-m}-1=\frac{m^{l+1}-m}{m-1}=\frac{m}{m-1}(m^{l}-1)≃m^{l},$$

while for *p*=1,

A 30 $$\sum_{k=1}^{l}(m-1)k\frac{m^{k}}{m^{k}-1}≃(m-1)\sum_{k=1}^{l}k=\frac{m-1}{2}\;l(l+1)≃\frac{m}{2}\;l(l+1),$$

hence entry from the start yields exponential learning times, but random entry only yields quadratic times, as reported in table 1.

**A.3. Extension to compound stimuli and continuous stimuli**

As mentioned in the main text, animals do not receive information about environmental states directly. Rather, they receive sensory information (including information from internal organs) that can, at any time, be described as a vector **x** of a certain size. An element *x*_*i*_ represents some feature of sensory information, which depends on what phenomena we wish to model and can range in granularity from the activity of a single receptor cell to high-level features such as the presence or absence of certain objects. We call **x** the stimulus vector.

We first generalize the decision-making equation, equation (2.2), so that a behaviour can be chosen given **x**. To this end, we introduce a set of vectors **v**_*B*_, one for each behaviour in the animal's repertoire, such that the value attributed to choosing *B* when sensing **x** is

A 31 $$V(B,\text{x})=\sum_{i}v_{B,i}x_{i}.$$

That is, the estimated value of choosing *B* is a weighted sum of the stimulus element values in **x**, with the elements of **v**_*B*_ as weights. We can thus adopt the following decision-making rule:

A 32 $$\mathrm{Pr}(B|\text{x})=\frac{e^{\beta V(B,\text{x})}}{\sum_{B^{\prime }}e^{\beta V(B^{\prime },\text{x})}}.$$

The learning rules in equations (2.4) and (2.5) are generalized similarly. Let *U*(**x**) be the primary value attributed to **x**, and let **w** be a vector such as the estimated value of **x** is

A 33 $$W(\text{x})=\sum_{i}w_{i}x_{i}.$$

We have then the learning equations

A 34 $$\Delta v_{B,i}=\alpha_{v}(U({\text{x}}^{\prime })+W({\text{x}}^{\prime })-v_{B,i})x_{i}$$

and

A 35 $$\Delta w_{i}=\alpha_{w}(U({\text{x}}^{\prime })+W({\text{x}}^{\prime })-w_{i})x_{i}.$$

Note the presence of the factor *x*_*i*_ in both equations. Its effect is to change more those elements of **v**_*B*_ and **w** corresponding to sensory elements with higher values. If, for example, *x*_*i*_=0, then neither *v*_*B*,*i*_ nor *w*_*i*_ would change.

The adoption of equations (A 31) and (A 33) corresponds to introducing two-layer artificial neural networks that respond to sensory input, rather than having a look-up table indexed by environmental state as used in the main text. These networks provide generalization in that similar stimulus vectors will receive similar valuations, and can also be sensitive to stimulus intensity if *x*_*i*_ is a continuous value increasing with stimulus intensity. Although simple, two-layer networks model successfully many aspects of stimulus generalization in animals [73,119]. If more complex processing of sensory information is desired, the functions *V* (*B*,**x**) and *W*(**x**) can be replaced, for example, by multi-layer or even recurrent neural networks.

We have not studied the extended model composed of equations (A 32), (A 34) and (A 35) in detail. The study of similar algorithms, however, has demonstrated robust abilities to approximate optimal behaviour [42,130,131].
